# Fatty Acid Profiles of *Leishmania major* Derived from Human and Rodent Hosts in Endemic Cutaneous Leishmaniasis Areas of Tunisia and Algeria

**DOI:** 10.3390/pathogens11010092

**Published:** 2022-01-14

**Authors:** Cyrine Bouabid, Yoshiki Yamaryo-Botté, Sameh Rabhi, Haifa Bichiou, Chaima Hkimi, Wafa Bouglita, Melek Chaouach, Naouel Eddaikra, Kais Ghedira, Lamia Guizani-Tabbane, Cyrille Y. Botté, Imen Rabhi

**Affiliations:** 1 Laboratoire de Parasitologie Médicale, Biotechnologies et Biomolécules (LR16IPT06), Institut Pasteur de Tunis, Université Tunis El-Manar, 13 Place Pasteur-BP74, Tunis 1002, Tunisia; cyrine.bouabid@pasteur.utm.tn (C.B.); samehrabhi@gmail.com (S.R.); haifabichiou@yahoo.fr (H.B.); wafabouglita@gmail.com (W.B.); mcmelek@msn.com (M.C.); lamia.guizani@pasteur.tn (L.G.-T.); 2 ApicoLipid Team, Institute for Advanced Biosciences, CNRS UMR5309, INSERM—National Institute for Health and Medical Research, Université Grenoble Alpes, INSERM U1209, 38000 Grenoble, France; yoshiki.botte-yamaryo@univ-grenoble-alpes.fr (Y.Y.-B.); or cyrille.botte@univ-grenoble-alpes.fr (C.Y.B.); 3 Laboratory of Bioinformatics, BioMathematics and Biostatistics, Institut Pasteur de Tunis, 13 Place Pasteur-BP74, Tunis 1002, Tunisia; hkimichaima27@gmail.com (C.H.); ghedirakais@gmail.com (K.G.); 4 Higher Institute of Biotechnology of Sidi Thabet, University of Manouba, Tunis 2050, Tunisia; 5 Laboratory of Eco-Epidemiology Parasitic Population Genetics, Pasteur Institute of Algiers, Algiers 16000, Algeria; neddaikra@yahoo.fr

**Keywords:** *Leishmania*, promastigote, human, rodent, lipids, fatty acids

## Abstract

Leishmaniasis is a protozoal vector-borne disease that affects both humans and animals. In the Mediterranean Basin, the primary reservoir hosts of *Leishmania* spp. are mainly rodents and canids. Lipidomic approaches have allowed scientists to establish *Leishmania* spp. lipid profiles for the identification of cell stage specific biomarkers, drug mechanisms of action, and host immune response. Using an in silico approach of global network interaction between genes involved in fatty acid (FA) synthesis followed by the GC-MS approach, we were able to characterize the fatty acid profiles of *L. major* derived from human and rodent hosts. Our results revealed that the lipid profile of *L. major* showed similarities and differences with those already reported for other *Leishmania* species. Phospholipids are the predominant lipid class. FA composition of rodent parasites was characterized by a lower abundance of the precursor C18:2(n-6). One of the rodent clones, which also expressed the lowest lipid abundance in PL and TAG, was the least sensitive clone to the miltefosine drug and has the lowest infection efficiency. Our findings suggest that the lipid composition variation may explain the response of the parasite toward treatment and their ability to infect their host.

## 1. Introduction

Leishmaniasis is an infectious neglected disease caused by a group of protozoan parasites (genus *Leishmania*) endemic in more than 98 countries and is considered as a public health problem. *Leishmania,* as a promastigote, is spread by the bite of sand flies and within mammalian hosts, it differentiates into amastigotes, the obligate intracellular parasite form. Due to their complex digenetic life cycle within their insect vector and animal host, *Leishmania* parasites can rapidly adapt to a new environment [1]. The outcome of the disease largely depends on the *Leishmania* species and host immunity. The latter is very important in disease development, although infections with *Leishmania* spp. lead to different clinical manifestations ranging from cutaneous lesions (CL) that heal spontaneously to the visceral form (VL), which can be fatal if left untreated [2]. In endemic areas, rodent species are considered as an important host reservoir to maintain the transmission of this disease [3]. In Tunisia and Algeria, the rodents *Psammomys obesus* and *Meriones shawi* are the most common host reservoirs for *Leishmania major* [4,5].

Genomic and metabolomic approaches have been successfully used to understand the parasite and the mechanisms that underline the dynamic interactions between the pathogen and its host, together sustaining parasite survival, propagation, and pathogenesis [6].

The lipidomic approach, one of the most dynamic sub-disciplines in metabolomics, provides qualitative and quantitative information about the lipid composition of a given biological sample (i.e., the lipidome) [7]. Lipid composition determines the biophysical properties of the parasite membrane [8] and regulates the structure, function, and anchoring of proteins to the membrane. More importantly, some lipid-based macromolecules act as virulence factors and play a role in the establishment of infection and the modulation of host immune response [9].

Lipidomics have been used for the identification of new lipid species in *Leishmania infantum* [10,11], and has also highlighted the lipid changes that occur during *Leishmania donovani* and *Leishmania infantum* promastigote differentiation into amastigote [12]. Lipidomics was also applied to demonstrate how lipid metabolism [13,14] impacts different parasite characteristics such as virulence and infectivity [15,16].

Complex lipids that are composed of fatty acids (FA) are considered as building blocks of most major lipid classes, and thus, biological membranes. Their abundance and composition can vary depending on different factors such as pathogen species, availability, de novo synthesis capacities, lipid recycling, physiological environment, and response to treatments. Lipid profiles have been investigated in several species of *Leishmania* such as *L. donovani, L. braziliensis, L. mexicana, L. tropica, L. amazonensis, L.* *tarentolae, L. enrietti*, and *L. infantum* [12,17,18,19,20]. Investigating lipids in *L. major* have mainly focused on identifying genes/proteins involved in one specific lipid pathway such as sphingolipid D4-desaturase/C4-hydroxylases [21] or alkyl dihydroxyacetonephosphate synthase *LmADS* and dihydroxyacetonephosphate acyltransferase *Lm*DAT were found essential for the synthesis of all ether glycerolipids [22,23]. In this organism, lipid detection was also used to identify the specificity of a specific metabolite such as ether phospholipids and glycosylinositolphospholipids and their function [24]. However, very few studies have reported the global lipid composition in *L. major.* A recent study demonstrated the function and importance of choline ethanolamine phosphotransferase (CEPT) in *Leishmania major*, assessing its importance for the de novo synthesis of PC and PE [25]. For glycerophospholipids, sterols, and sphingolipids, studies have focused on elucidating the balance between promastigote *L. major* de novo synthesis and amastigote lipid salvage and remodeling [26].

Given the crucial role of lipids and their variability in both promastigote and amastigote forms of *L. major*, lipidomic profiles of parasites deriving from different hosts are missing, although host and parasite nature have a massive impact on lipid composition and homeostasis.

In fact, the parasite depends on its host to scavenge/acquire lipids, and the multiplicity in hosts and their metabolic profiles could cause an important change in lipid profiles of the parasites. Such changes are the reflection of different parasite metabolic pathways to maintain lipid homeostasis, hence, involving different proteins with various degrees of importance depending on the host and its conditions.

In this study, we aimed at establishing the lipid profile of the *L. major* parasite and comparing FA composition in parasites from two different hosts (human and rodent) to evaluate differences in lipid profiles in parasites from these host reservoirs, which could affect treatment efficiency. These differences may help to develop a new strategy for the control and prevention of the emergence of this disease in reservoirs.

Here, we performed an in silico analysis demonstrating the predominance of polyunsaturated fatty acids in *L. major* promastigote. The mass spectrometry based lipidomic approach confirmed that this class of FA was predominant among the different *Leishmania* strains used in this study. GC-MS also revealed differences in FA composition in the *L. major* promastigotes, especially in C18:2. The difference among the strains was mainly in the abundance rather than the composition of FA, which was overall conserved. These results suggest that lipid composition variation may explain the response of the parasite toward treatment and their ability to infect the host.

## 2. Results

### 2.1. In Silico Leishmania major Genes and Related Lipids Compounds Interaction Network

**Figure 1:**
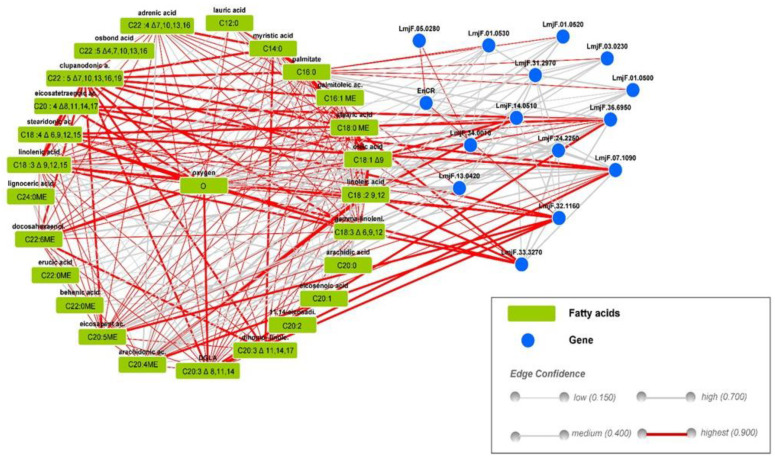
*Leishmania major* gene–compound interaction network. Blue nodes represent genes. Fatty acid compounds are highlighted in green; red lines represent the highest confidence in gene compound interaction (edge confidence = 0.900).

We first explored all *Leishmania major* genes involved in the fatty acid synthesis pathways and performed an in silico analysis using the StitchDB database for interaction networks of chemicals, genes, and protein analysis (Figure 1).

TritrypDB kegg pathways enrichment of the 39 *Leishmania major* genes implicated in lipid pathways [27] revealed that most of the identified genes (15 out 39) belonged to the biosynthesis of unsaturated fatty acid pathway (https://tritrypdb.org/tritrypdb/app/record/pathway/KEGG/ec01040, accessed on 5 May 2021). We next used KeggDB to investigate both genes and compounds involved in the lma01040 Biosynthesis of unsaturated fatty acids pathway in the *Leishmania major* promastigote (https://www.genome.jp/entry/pathway+lma01040, accessed on 5 May 2021) species. Finally, the gene list was loaded into StitchDB to generate a *Leishmania major* gene–compound interaction network highlighted in Figure 1.

According to our findings, these genes are either involved in the long chain fatty acid CoA synthase, the elongation pathway, or coding for desaturase proteins. Some of these genes were biologically validated for their function such as LmjF.24.2250, LmjF14.0510 coding for Δ9 type I desaturase, LmjF.32.1160 Δ6 desaturase, LmjF.33.3270 Δ12 desaturase, LmjF07.1090 Δ5 desaturase, and LmjF31.2970 acetyl CoA carboxylase (ACC).

This analysis demonstrates the diverse interaction between genes involved in fatty acid synthesis and fatty acid compounds. Indeed, LmjF14.0510 coding for Δ9 type I desaturase highly interacted with C18:1Δ^9^; this latter also interacted with LmjF.33.3270 Δ12 desaturase, LmjF.32.1160 Δ6 desaturase (Elo6), and C18:3n-6 and C20:3n-6. We also noticed a strong connection between LmjF07.1090 Δ5 desaturase and C20:3n-6 as well as C20:4n6. These in silico results indicate the predominance of polyunsaturated fatty acid in the *Leishmania major* promastigote.

### 2.2. Mass Spectrometry-Based Lipidomic Analyses Reveal the Total FA and Sterol Composition of Different L. major Clones Differing from Their Host Nature

To establish a lipid profile for the different *Leishmania major* promastigote clones, all parasites were grown until reaching the stationary growth phase; after a quenching step, total lipids were extracted and analyzed based on the fatty acid abundance using GC-MS.

**Figure 2:**
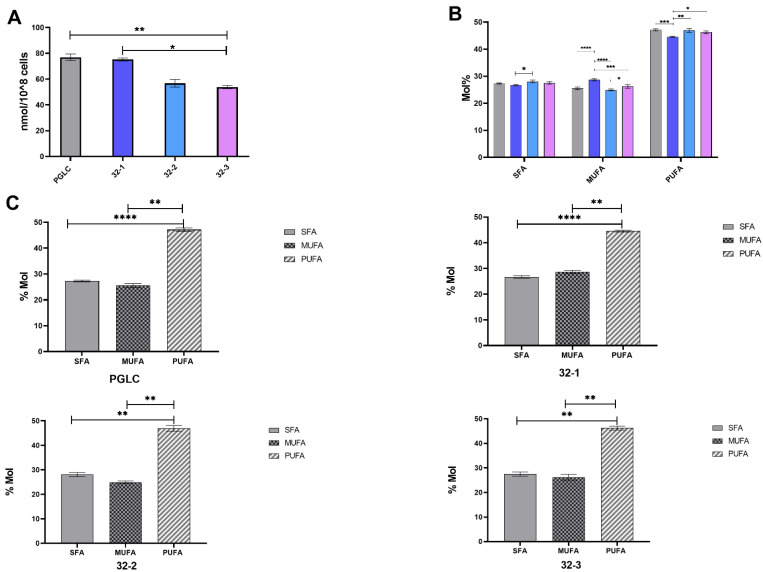
Total lipid abundance among the *Leishmania major* parasites isolated from human and rodent. (**A**) Total lipid abundance among human (PGLC), rodent isolates 32-1, 32-2, 32-3. (**B**) Relative abundance of saturated fatty acid (SFA), mono unsaturated fatty acid (MUFA), and poly unsaturated fatty acid (PUFA). (**C**) Different distribution of fatty acid class in each *Leishmania major* promastigote. PGLC (grey), 32-1 (blue), 32-2 (light blue), and 32-3 (purple). Data are the means ± SEM of three independent determinations. (ANOVA test) * *p* ≤ 0.05, ** *p* ≤ 0.01, *** *p* ≤ 0.0001, **** *p* < 0.000004 significant differences between the different *Leishmania major* promastigotes. GraphPad Prism 8.

The total lipid (fatty acid) content (Figure 2A) showed differences between human and rodent parasites and among the rodent origin clones. While rodent clone 32-1 had similar lipid abundance as the human origin *L. major* (75, 77 nmol/10^8^ cells, respectively), clone 32-2 (56.7 nmol/10^8^ cells) and 32-3 (53.8 nmol/10^8^ cells) showed significantly lower abundance. The major fatty acids of the total lipid identified were C12:0, C14:0, C16:0, and C18:0 for the saturated fatty acid species. The monounsaturated fatty acids were mainly constituted by oleic acid C18:1 (Table 1).

Finally, polyunsaturated fatty acids are essentially composed of C18:2, C18:3, C22:6, C20:2, and C20:3n-3/n-6. PUFAs are statistically the predominant fatty acid class in all our clones. However, significant differences were observed between the different clones regarding the abundance of PUFA (Figure 2B,C). These findings are in line with the in silico analysis (Figure 1).

**Figure 3:**
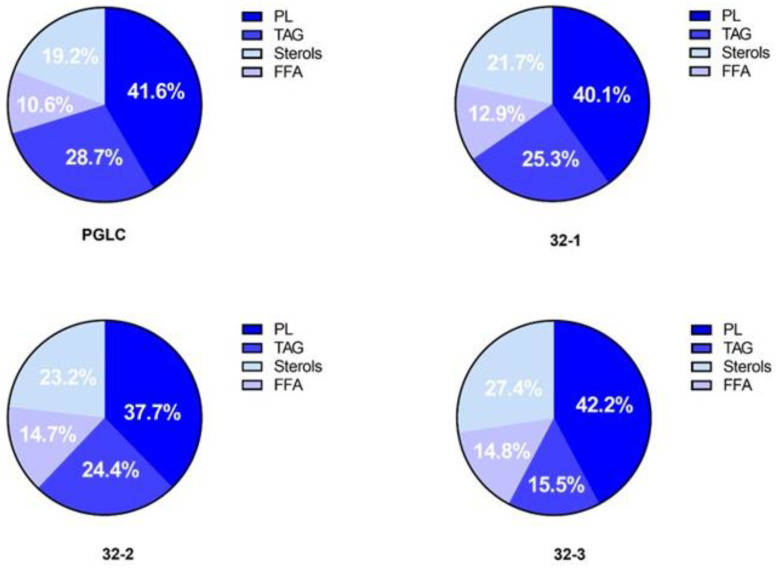
Major lipid class relative abundance in *Leishmania major* promastigotes isolated from human and rodent. Molar percentages (Mol%) were calculated in relation to the total lipids for each strain. PL (phospholipids), TAG (triacylglycerol), sterols, and free fatty acids (FFA). Human isolate: PGLC, rodent isolates: 32-1, 32-2, 32-3.

Based on the variation identified in terms of total lipid (total fatty acid content extracted from total lipid pools) content, we investigated the abundance of phospholipids (PLs), free fatty acids (FFAs), and neutral lipids (i.e., triacylglycerol (TAG) and sterols (cholesterols and ergosterol) in all of our *L. major* promastigotes. The total lipids were separated using TLC 1D and analyzed by GC-MS. Our results (Figure 3) show that for all *Leishmania* parasites, PLs were the most predominant lipid class ranging from 37.7% to 42.2%, followed by TAG, demonstrating a relative abundance ranging from 15.5 to 28.7%. Sterols were also detected with a relative abundance comprised between 19.2% and 27.4% and finally, FFA represented a lower portion with 10.6% for PGLC and 14.8% for 32-3.

**Figure 4:**
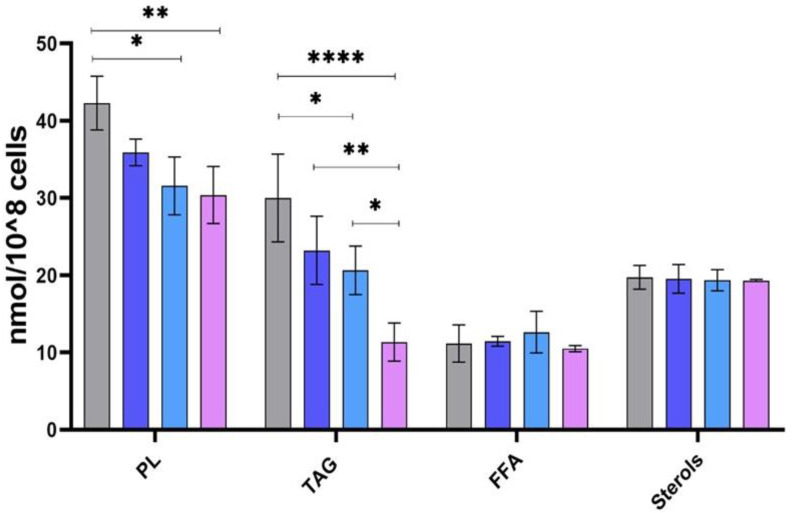
Abundance of PL, TAG, sterols, and FFA in *Leishmania major* promastigotes isolated from human and rodent. Abundance in nmol/10^8^ cells for human isolate PGLC (grey), rodent isolates: 32-1 (blue), 32-2 (light blue), and 32-3 (purple). Data are the means ± SEM of three independent determinations. (ANOVA test) * *p* ≤ 0.05, ** *p* ≤ 0.01, **** *p* < 0.000004 significant differences between the different *Leishmania* promastigotes.

No statistical differences were observed for the abundance of sterols and FFA between the different clones (Figure 4). However, PL and TAG expressed significant changes in their abundance. These changes were reflected by the significant difference in PL and TAG content between the human and two rodent clones (32-3 and 32-2). We also noticed some significant changes between the rodent clones in the TAG fraction and a significant trend of less TAG abundance in the rodent strains. Clone 32-3 had the lowest abundance of PL and TAG. However, PL and TAG expressed significant changes in their abundance. These changes are reflected by the significant difference in PL and TAG content between the human and two rodent clones (32-3 and 32-2).

We also noticed some significant changes between the rodent clones in the TAG fraction and a significant trend of less TAG abundance in the rodent strains. Clone 32-3 had the lowest abundance of PL and TAG.

### 2.3. GC-MS Analyses Reveal Differences in the FA Composition of the Different Clones

**Figure 5:**
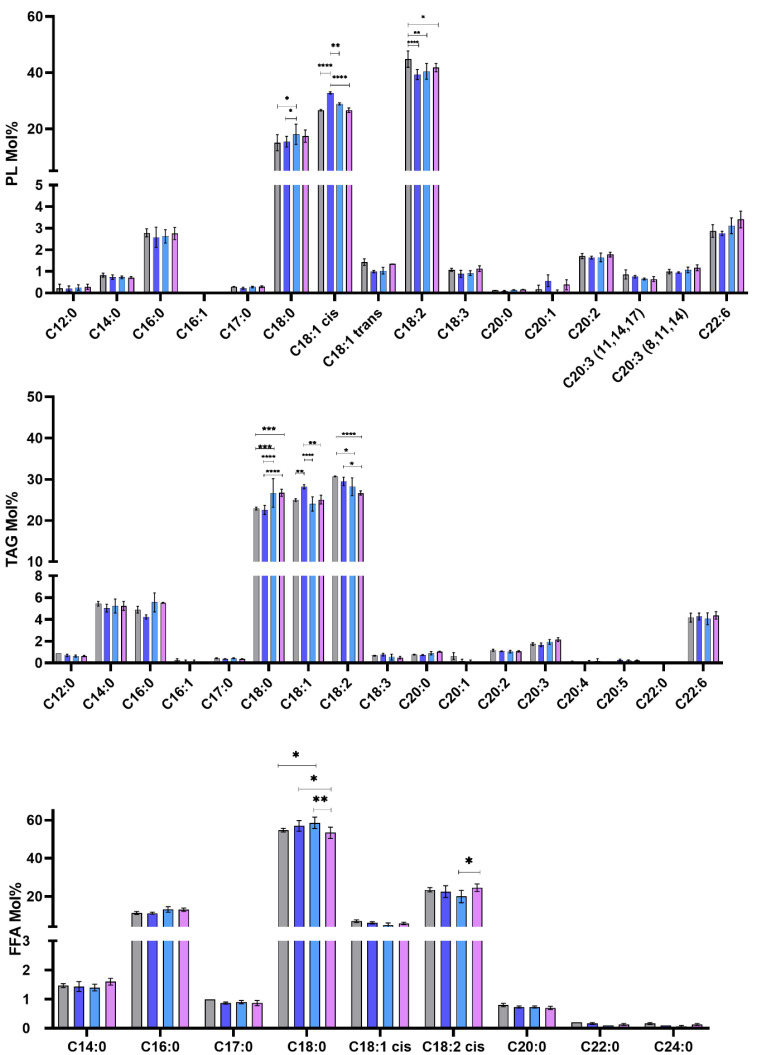
Fatty acid composition of the PL, TAG, and FFA fractions in *Leishmania major* promastigotes isolated from human and rodent. Molar percentages (Mol%) were calculated relative to the total lipids for each strain. PL (phospholipids), TAG (triacylglycerol), sterols, and free fatty acids (FFA). PGLC (grey), 32-1 (blue). 32-2 (light blue) and 32-3 (purple). Data are the means ± SEM of 3 independent determinations. (ANOVA test) * *p* ≤ 0.05, ** *p* ≤ 0.01, *** *p* ≤ 0.0005, **** *p* < 0.000004 significant differences between the different *Leishmania* promastigote.

Since the relative abundance varied among the strains and clones, we further investigated the fatty acid composition of the separated PL, TAG, and FFA. As shown in Figure 5, TAG was distinguished by the presence of an elevated abundance of myristic acid (C14:0) compared to PL and FFA. Palmitic (C16:0) and stearic (C18:0) acids were eminently present in the FFA fraction.

In addition, among the lipid classes, our data also showed that there was an outstanding difference between the human and rodent isolates regarding fatty acid composition. The first remarkable variations were related to the abundance of several fatty acids mainly, C18:0, oleic acid (C18:1), and linoleic acid (C18:2). The human isolate exhibited a higher abundance of C18:2 fatty acids than the rodent clones, especially in PL and TAG. In the 32-2 and 32-3 clones, the reduction in C18:2 was compensated by the other polyunsaturated acids. Regarding 32-1, the compensation was demonstrated by the increase in C18:1. The 32-1clone can therefore be characterized by the abundance of the latter. This suggests that there is a distinct difference in the fatty acid metabolism within the same isolate as it could be related to the expression of the Δ12 enzyme responsible for the conversion of C18:1 into C18:2.

**Figure 6:**
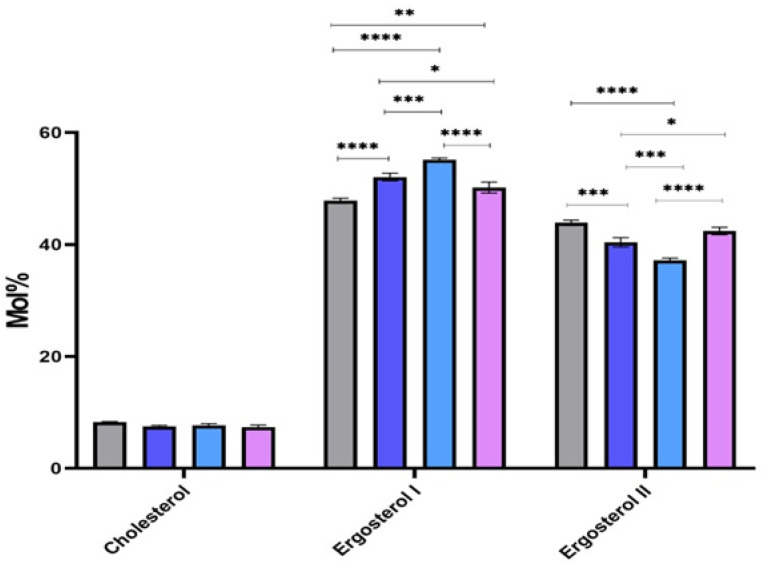
Sterols identified by gas chromatograph-mass spectrometry (GC-MS) in *Leishmania major* promastigotes isolated from human and rodent. Molar percentages (Mol%) were calculated in relative to the total lipids for each strain. PGLC (grey), 32-1 (blue), 32-2 (light blue), and 32-3 (purple). Data are the means ± SEM of three independent determinations. (ANOVA test) * *p* ≤ 0.05, ** *p* ≤ 0.01, *** *p* ≤ 0.0003, **** *p* < 0.000004 significant differences between the different *Leishmania* promastigote.

Regarding sterols, we were able to detect cholesterol, ergosterol I, and ergosterol II, an isomer of ergosterol I (Figure 6). Our data demonstrate the conspicuous presence of ergosterol and its isomer as a major sterol in all our *Leishmania* parasites. We noticed a stable abundance of cholesterol in all strains, while a fluctuation of the abundance of ergosterol and its isomer was noticed. Indeed, 32-1/2/3 revealed more ergosterol than the *L. major* isolated from human, whereas PGLC showed more abundancy of the ergosterol isomer.

### 2.4. Neutral Lipids and Lipids Droplet Content

Since we measured a reducing trend in the presence of TAG in the rodent clones, we thus decided to further investigate the lipid droplet (LD) content in the different clones. We used Nile Red to visualize the parasite lipid droplet and their neutral lipid content.

**Figure 7:**
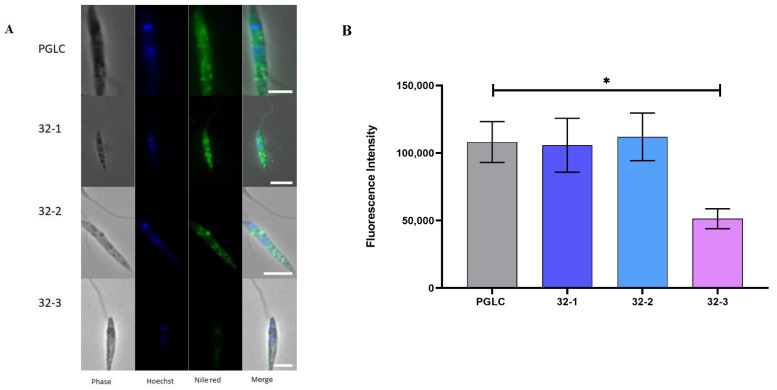
Lipid distribution analysis in *Leishmania major* promastigotes isolated from human and rodent. (**A**) Fluorescence visualization by APOTOME microscope using Nile Red staining. (**B**) Nile Red intensity fluorometric quantification. PGLC (grey), 32-1 (blue), 32-2 (light blue), and 32-3 (purple). Data are the means ± SD of two independent determinations. (Student’s t-test) * *p* ≤ 0.05. significant differences between the different clones.

As shown in Figure 7A, there were variations in the fluorescence intensity of LD among the different isolates, which suggests their heterogeneity regarding the abundance of neutral lipids. However, this fluorescence intensity variation was statically significant only between PGLC and 32-3. (Figure 7B). These results confirm our observations regarding the abundance of TAGs in this clone (Figure 4).

### 2.5. In Vitro Assay for Drug Susceptibility and Host–Pathogen Infectivity Ratio and Lipid Content Correlation

Since there was a difference in the lipid profile and in the Nile Red staining for lipid droplets, we investigated whether there was an alteration of the parasite’s drug sensitivity and their infectivity in their correlation to the lipidomic profiles. We used an MTT assay to calculate the IC_50_ for miltefosine and Sb(III) drugs. Our results, summarized in Table 2, show that all our *L. major* strains expressed IC_50_ values ranging from 4.71 to 6.09 µg/mL for Sb(III) and from 9.93 to 13.24 µM for miltefosine. These data suggest that all parasites are sensitive to Sb(III) and miltefosine, except for the 32-3 rodent isolate, which expressed an IC_50_ slightly higher than the threshold for the resistance strains to miltefosine. Moreover, we assessed the in vitro macrophage infectivity, 24 h post infection. As shown in Table 2, clone 32-3 expressed the lowest infectivity (37%) percentage compared to the other isolates.

These results suggest that lipid composition variation may explain the parasites’ response toward treatment and their ability to infect their host.

## 3. Discussion

In this study, we aimed at obtaining a detailed descriptive lipid profile of *L. major* and tried to detect changes in this class of metabolite between the different clinical isolates of *L. major* promastigotes from Tunisia and Algeria obtained from either human or rodent lesions.

To achieve our goal, a lipidomic approach was used based on GC-MS analysis. This approach proved its relevance through various findings demonstrating the importance of lipids in Trypanosomatidea and their implications on different functions ranging from their involvement in virulence to their use as biomarkers [7]. For years, fatty acid acquisition and de novo biosynthesis in Trypanosomatidea such as *Leishmania* have been controversial issues among scientists. This synthesis pathway in Trypanosomatidea is divergent from the one of their hosts (mammals), and their genome study demonstrates the presence of the FAS II pathway and elongase system [27].

Our in silico analysis identified 25 FA that showed network interaction among genes involved in FA synthesis and fatty acid compounds. Experimentally, we were able to detect 16 byproducts using GC-MS. Among these were the saturated C18:0 and the monounsaturated C18:1, which suggest the activation of Δ9 desaturase. We also detected, by GC-MS, C22:6 (n-3), which suggests the Δ4 desaturase activation. In addition, we showed the predominance of polyunsaturated fatty acids (PUFA) in all our isolates, suggesting the activation of Δ5, Δ6, desaturases, and Δ6 elongase, which implies the complete activation of the polyunsaturated FA pathway. These results are in accordance with a previous study showing the activity of Δ4, Δ5, and Δ6 in Trypanosomatidae [28].

Previous studies from the 60s [18,19,29] have tried to determine the lipid profile of different *Leishmania* species using different techniques and platforms. In our analysis involving *Leishmania major,* we found that the principal fatty acids of the total lipids within the different classes were C14:0, C16:0, C20:2n-6, C20:3n-6, and C22:6n-3. Despite the variety of techniques and/or platforms used to identify FA composition, similar findings have been previously reported for *L. tropica, L. mexicana, L. donovani, L. amazonensis,* and *L*. *tarentolae* [18,19,20,29,30]. We noted the absence of C22:5 (n-6), detected in *L. donovani* and *infantum* [12], the absence of C18:3, found abundant in *L. donovani* PC [31] as well as C18:4 and C20:4 (n-3) not detected in *L. donovani* and *L. infantum* [12]. We were, however, able to detect the C22:6 end product of the pathway. These discrepancies could reflect differences in the abundance of these compounds between the different *Leishmania* species and/or in the lipidomic platform used.

In Tunisia and Algeria, *L. major* represents the causative agent of cutaneous leishmaniasis (CL) [32,33]. CL is a dynamic disease involving specific vectors, reservoirs, and host reservoirs. In North Africa, *Psammomys* is the main reservoir host of *L. major* and the source of epidemics in this region [34]. The reservoir host is crucial for the maintenance of the parasite [35], in contrast to the incidental host, which is inconsequential for the persistence of the disease [36]. Previous studies on Trypanosomatidea have shown that their fatty acid composition depends on their environment [37,38]. The dynamic interaction between the parasite and the host reservoir that compete for the same metabolic resources could explain the differences in the lipid profile observed among our clones. However, this difference mainly concerns the abundance rather than the composition, which was overall conserved. Indeed, despite the similar culture conditions, the abundance of total lipid content, but also phospholipids and TAGs, was lower in rodent clones. At the level of FA composition, these differences mainly concerned the abundance of C18:2, which was lower in rodent isolates.

Concerning sterols, we noted the presence of ergosterol, synthesized by kinetoplast, unlike mammalian cells [39]. We also noticed the presence of a stable amount of cholesterol, most likely supplied by the culture medium given the inability of this parasite to synthesize it [10].

When we assessed the infectivity of our clones, the clones isolated from rodents were the least efficient. This is especially true for clone 32-3, expressing 37% of infectivity. The latter expressed the lowest abundance of phospholipids. These cellular membrane components play a crucial role in the infectivity and virulence of the parasite [40]. It is therefore reasonable to think that a decrease in their abundance would change the physico-chemical properties of the membrane, and thus, the function that depends on it. Indeed, different lipid containing molecules such as LPG are surface virulence factors. In addition, plasmenylethanolamine (PLE), an essential component of PL, increases as the parasite moves into a stationary phase rich in infective promastigotes [40].

Lipid composition is crucial for membrane fluidity and may also influence drug–membrane interactions. Our strains expressed different susceptibility degrees to drugs and expressed Sb(III) IC_50_ values ranging from 4.71 to 6.09 µg/mL and are thus, according to Maes et al. (2002), susceptible to antimonies [41]. The reservoir host, associated with the geographic distribution (localization), could explain these differences in susceptibility. Indeed, Fernandez et al. demonstrated that clinical isolates from *L. Viannia*, the causative agent of CL in Colombia, isolated from different geographical regions, responded differently to Sb(V) and miltefosine [42]. Some correlations were made between the acquisition of Sb(III) resistance and variation in *Leishmania* lipid profile, leading to the identification of biomarkers [20]. In fact, sensitive clones to antimonies express a higher amount of C18:1 Δ^9^ (desaturase) and a decrease in C20:4; this profile matched that of clone 32-1, which showed an IC_50_ equivalent to 5.47 µg/mL of Sb(III).

Nonetheless, our understanding of the specific lipid changes during the development of antimony resistance is still limited. Gutierrez Guarnizo et al. (2021) reported, on resistant phenotypes of *L. tropica*, a downward trend in phospholipids, particularly in phosphatidylcholine and a shift to an upward trend in TAGs and long-chain fatty acids in susceptible phenotypes [43]. Among the studied clones, clone 32-3, which was the least sensitive to Sb(III) contained the lowest content of phospholipids as well as TAG and its long-chain fatty acids. 32-3 was also the only clone expressing an IC_50_ of 13.20 µM for the miltefosine (MIL) drug that targets phospholipids and proved its leishmanicidal activity [44,45], but it is not used in North Africa. This value was slightly higher than the threshold determined in a study conducted by Escobar in 2002, who reported that sensitivity to MIL is species dependent [46]. The most sensitive species was *L. donovani* and the least sensitive was *L. major* with an IC_50_ ranging from 4.8 to 13.24 µM. As described for the *L. dononvani* resistant strain, the 32-3 clone expressed the lowest amount of C18 fatty acids [47].

Regarding the crucial role played by lipids in Trypanosomatidae infectivity [40] and resistance to the antileishmanial drug [7], our study has provided interesting insights on the total lipid distribution as well as fatty acid composition in *L. major* from two key reservoir hosts.

Further studies are required to identify the FA classes that could trigger the host immune response toward an M1 (classically activated) or M2 (alternatively activated) phenotype. The macrophage polarization is crucial for the outcome of the disease and could be associated with mechanisms of drug resistance as well as with cell growth efficiency and infectivity in *L. major.*

Further extensive research is necessary to complete our lipidomic profile by investigating lipid changes of *Leishmania* infected macrophages and characterizing the immune response of the mammalian host cells either infected by the parasite or treated by *Leishmania* derived lipids.

## 4. Materials and Methods

### 4.1. Parasite Culture

The parasites used in our study belong to the *L. major* species. They differ in their geographic origin and the origin of the lesions from which they were isolated.

*L. major* PGLC 94 is a reference Tunisian strain MHOM/TN/95/GLC94 zymodeme MON-25 originating from a Tunisian strain collected in 1994 at El Guettar, southern Tunisia and isolated from human. PSAM/DZ/2006/LIPA 32/06 zymodeme MON-25 was kindly provided by Dr Naouel Eddaikra from a reference laboratory (Laboratory of Eco-epidemiology Parasitic Population Genetics, Pasteur Institute of Algiers). This strain was isolated from Tougourt in 2006 from *Psamommys Obeus.* These parasites, purified from rodent, grown on NNN (Nicolle–Novy–MacNeal) medium and conserved in liquid nitrogen, were thawed, and limited dilution was realized to obtain the three different clones designated as 32-1/32-2/ and 32-3. To maintain the virulence of the Tunisian strain isolated from humans, 10^7^ promastigotes were inoculated in the footpad of Balb/c mice. The parasites used in all the experiments were promastigotes that came from the amastigotes purified from the lesions and cryopreserved after three passages. The parasites purified from rodent were grown on NNN medium and conserved in liquid nitrogen.

*Leishmania major* promastigotes were grown in the culture flask at 26 °C in RPMI 1640 (Sigma), supplemented with 10% heat-inactivated fetal bovine serum (Gibco), 2 mM L-glutamine, penicillin (100 U/mL) (Sigma), and streptomycin (100 μg/mL) (Sigma). The parasites were isolated during their stationary phase by centrifugation at 2117 g for 10 min.

### 4.2. Bioinformatic Analysis

List of *Leishmania major* characterized and hypothetical proteins involved in transport, synthesis, and modification of fatty acids were retrieved from Table 1 in the review by Antonio D. Uttaro (https://pubmed.ncbi.nlm.nih.gov/24726787/, accessed on 5 May 2021) [27]. In their review, Uttaro and co-workers listed all TriTrypDB gene names and accession numbers of characterized and hypothetical proteins involved in the transport, synthesis, and modification of fatty acids. The latter list containing 39 parasite genes was uploaded into the TritrypDB database to identify enriched Kegg pathways associated with these genes (https://tritrypdb.org/tritrypdb/app/workspace/strategies/323086893/425130523, accessed on 5 May 2021; https://academic.oup.com/nar/article/44/D1/D457/2502600, accessed on 5 May 2021). The most significant pathway including the highest number of genes from the gene list was searched and visualized in KeggDB (https://www.ncbi.nlm.nih.gov/pmc/articles/PMC102409/, accessed on 5 May 2021) and all related components including genes and compounds were retrieved. This later list was uploaded into StitchDB (https://academic.oup.com/nar/article/44/D1/D380/2503089, accessed on 5 May 2021) for network generation and component *Leishmania major* gene interaction visualization.

### 4.3. Parasite Harvesting

The parasites were seeded at 3 × 10^6^/mL and collected six days later. For optimum lipid recovery, we used an extraction based on chloroform/methanol. For all the GC-MS analysis, internal and external triplicates were realized.

Parasites with the medium in the culture flask were metabolically quenched by rapid chilling in a dry ice-ethanol slurry bath and then centrifuged down at 4 °C.

The cell pellet was suspended in ice-cold phosphate buffered saline (PBS) (Sigma), counted using a Mallassez chamber for 1 × 10^8^ cells and washed with PBS.

### 4.4. Lipid Extraction

Total lipids were extracted as described previously in [48]. Briefly, parasite lipids were extracted in chloroform/methanol (1:1, *v/v*) containing FFA (free fatty acids) C13:0, (10 nmol), PC 21:0 (20 nmol), and stigmasterol (Avanti Polar lipids) as internal standards for extraction and normalization to the original abundance. A bath sonicator was used to dissociate the precipitation of the pellet for better lipid extraction. The samples were incubated overnight at +4 °C. After centrifugation, the supernatant was pooled into another vial, and lipids were further extracted from the parasite pellet with chloroform/methanol (2:3; *v/v*) (Sigma).

### 4.5. Total Lipid Analysis

Fifty µL from each sample was dried using a vacuum pump and derivatized using trimethyl sulfonium hydroxide (TMSH) (Machery-Nagel) to obtain fatty acid methyl ester (FAME) and N,O-bis trimethylsilyl)trifluoroacetamide with trimethylchlorosilane (BSTFA-TMCS) (Sigma) to obtain sterol-TMS. The resulting metabolites were quantified by GC-MS (gas chromatography-mass spectrometry) (Agilent 7890B-5977A), according to the method described by [48,49] and their identification was based on GC retention time and mass spectra compared with authentic FA standards (CRM47885, Sigma). The concentration of FAMEs and sterols were quantified after initial normalization to different internal standards as well as the cell numbers. All samples were taken in triplicate from independent cultures.

### 4.6. Thin Layer Chromatography for Neutral Lipids

Total lipid was resuspended in butanol and separated by high performance thin-layer chromatography (HPTLC, Silica gel 60, Merck), as described previously [50]. HPTLC was migrated with the solvent system hexane/diethyl ether/formic acid (40:10:1, *v/v/v*, Sigma) and revealed under UV light after spraying with purimuline (1 × 10^−3^%, Sigma) solution in 80% acetone. Different lipids were identified by comparison with an authentic standard spotted on the same plate. The spots correlating to phospholipids, DAG, sterols, free fatty acids, and TAG were scraped off. An internal standard (C15:0 1 nmol) (Avanti Polar lipids) was added and the methanolysis was carried out by hydrogen chloride solution. All samples were incubated at 85 °C for 3 h (oven) [50].

A second extraction was realized by adding hexane (Sigma) and water. The supernatant was transferred. For sterols, the derivatization was made by adding BSTFA-TMCS (Sigma) and for the rest of lipids, hexane was added. All the samples were analyzed using GC-MS (Agilent). For lipidomic analysis, three independent experiments were performed in triplicate for each sample.

### 4.7. Detection of Lipid Droplets Using Nile Red

A sample of 1 × 10^7^ parasites were fixed in 4% paraformaldehyde + 6 × 10^−4^% glutaraldehyde for 30 min (Electron Microscopy Science), permeabilized with 0.25% Triton (Sigma) for 10 min with PBS wash after each step. Then, cells were stained with 5 µg/mL of Hoechst (Life Technologies, Carlsbad, CA, USA) for DNA for 30 min and with 10 µg/mL Nile Red (Sigma) for lipid droplets for 1 h. The cells and fluorescence were visualized and measured using an APOTOME microscope AxioImager Z1 equipped with the CCD Zeiss camera (excitation: 450–500 nm; emission: 528 nm). Two independent experiments were performed.

### 4.8. In Vitro Drug Sensitivity Assay

The drug susceptibility of the two strains was assessed using the MTT (Sigma) assay. A sample of 2 × 10^6^ parasites were seeded in 96-well plates and treated with the recommended concentration of Sb(III) (80 µg/mL–5 µg/mL) and miltefosine (40 µM–2.5 µM) (Sigma) [41]. The plates were incubated for 72 h at 26 °C and 0.5 mg/mL of MTT were added to each well. After 4 h of incubation, OD was determined at 540 nm. IC_50_ was determined using GraphPad Prism 8. Three independent experiments were performed.

### 4.9. Parasite Infectivity

THP-1 cells (ATCC) were differentiated into macrophages by 24 h incubation with phorbol 12-myristate 13-acetate 20 ng/mL (PMA, Sigma) at 37 °C and 5% CO_2_. The cells were washed and incubated in RPMI medium for 24 additional hours. To infect macrophages, stationary parasites were incubated with the differentiated THP1 cells at a ratio of 1:10. After 24 h, the cells were washed three times with PBS 1x to remove extracellular parasites. The parasite infectivity was evaluated using the Giemsa staining procedure [41]. The percentage of infected cells was calculated on 100 cells. Two independent experiments were achieved.

### 4.10. Statistical Analysis

For all the statistical analysis performed on lipidomic data, we used (ANOVA) analysis of variance, allowing the multiple comparisons between the different *L. major* promastigote: a *p* value smaller than 0.05 (*p* < 0.05) was considered significant. For fluorescence intensity, ImageJ was used, followed by the student’s t-test. All the graphs and figures were made using GraphPad Prism 8.

## Figures and Tables

**Table 1:** Fatty acid composition of total lipids in the different *L. major* promastigotes.

Fatty Acid Composition		*L. major* Reference Isolates			
FA (Mol%)		PGLC	32-1	32-2	32-3
SFA	C12:0	1.4 ± 0.2	1.6 ± 0.2	2 ± 0.2	1.9 ± 0.5
	C14:0	3.5 ± 0.2	3.4 ± 0.2	3.2 ± 0.3	3 ± 0.3
	C16:0	3.1 ± 0.2	2.7 ± 0.1	3 ± 0.4	3. ± 0.2
	C17:0	0.5 ± 0.0	0.4 ± 0.0	0.5 ± 0.1	0.3 ± 0.1
	C18:0	18.3 ± 0.3	18.1 ± 0.4	19 ± 0.9	18.8 ± 0.2
	C20:0	0.5 ± 0.0	0.5 ± 0.0	0.5 ± 0.0	0.5 ± 0.1
	C22:0	0.0	0.0	0.1 ± 0.1	0.0
	C24:0	0.1 ± 0.1	0.1 ± 0.1	0.1 ± 0.0	0.1 ± 0.1
	Total	27.4	26.8	28.4	27.6
MUFA	C16:1	0.1 ± 0.1	0.2 ± 0.1	0.0	0.1 ± 0.1
	C18:1 cis	24 ± 0.3	26.9 ± 0.8	23.1 ± 1.0	24.3 ± 0.9
	C18:1trans	1.5 ± 0.4	1.6 ± 0.2	1.8 ± 0.7	1.8 ± 0.5
	C20:1	0.0	0.0	0.0	0.0
	C22:1	0.0	0.0	0.0	0.0
	C24:1	0.0	0.0	0.0	0.0
	Total	25.6	28.7	24.9	26.2
PUFA	C18:2 (n-6)	38.8 ± 0.6	36.5 ± 0.1	38.1 ± 1.3	36.9 ± 1.4
	C18:3 (n-6)	1.1 ± 0.1	1.2 ± 0.2	1.2 ± 0.1	1.1 ± 0.2
	C20:2 (n-6)	1.5 ± 0.0	1.4 ± 0.1	1.5 ± 0.2	1.5 ± 0.1
	C20:3 (n-3)	0.6 ± 0.1	0.6 ± 0.2	0.9 ± 0.1	0.8 ± 0.1
	C20:3 (n-6)	1.3 ± 0.1	1.2 ± 0.1	1.4 ± 0.1	1.4 ± 0.1
	C20:4 (n-6)	0.0	0.0	0.0	0.2 ± 0.2
	C20:5(n-3)	0.0	0.0	0.0	0.1 ± 0.1
	C22:2	0.0	0.0	0.0	0.0
	C22:6 (n-3)	3.7 ± 0.1	3.5 ± 0.2	3.9 ± 0.2	4.1 ± 0.4
	Total	47.0	44.4	47	46.1

Fatty acid composition was determined by GC analysis as described in the Materials and Methods. Data are means of three independent experiments ± SEM. Human isolate PGLC, rodent isolates 32-1, 32-2, 32-3. FA: Fatty acids, SFA: Saturated fatty acids, MUFA: Mono unsaturated fatty acids, PUFA: Poly unsaturated fatty acids. Mol%: Molar percentage.

**Table 2:** IC_50_ of the different reference drugs and infectivity percentage in *Leishmania major* promastigotes isolated from human and rodent.

	*L. major* Reference Isolates			
	PGLC	32-1	32-2	32-3
Sb(III) µg/mL	4.71 ± 0.5	5.47 ± 0.34	3.62 ± 1.04	6.09 ± 0.16
Miltefosine µM	9.93 ± 3.7	12.26 ± 2.19	10.29 ± 0.03	13.24 ± 1.39
Infectivity	53% ± 13.4	40% ± 2.8	47% ± 4.9	37% ± 9.1

IC_50_ of Sb(III) and miltefosine. Human isolate: PGLC, rodent isolates: 32-1, 32-2, 32-3. Infectivity percentages were calculated according to infected cells and non-infected cells. The means ± were determined from three independent experiments for the MTT assay and two independent experiments for parasite infectivity.

## Data Availability

Authors can confirm that all relevant data are included in this paper.
